# Effect of high pressure treatment and short term storage on changes in main volatile compounds of Chinese liquor

**DOI:** 10.1038/s41598-017-17549-x

**Published:** 2017-12-08

**Authors:** Menglong Xu, Songming Zhu, Hosahalli S. Ramaswamy, Yong Yu

**Affiliations:** 1 College of Biosystems Engineering and Food Science, Zhejiang University, Hangzhou, 310058 China; 2 Key Laboratory of Equipment and Informatization in Environment Controlled Agriculture, Ministry of Agriculture, Hangzhou, 310058 China; 3 Department of Food Science, McGill University, St-Anne-de-Bellevue, QC H9X 3V9 Canada

## Abstract

Changes in main volatile compounds of Chinese liquor after high pressure (HP) treatment and following short term storage were investigated. 400 MPa-15 min & 400 MPa-30 min were applied to young liquor. Results from gas chromatography (GC) analysis revealed decreasing trends in alcohols, aldehydes and ethyl acetate immediately after HP treatments, which was in consistent with those in natural aging process; but no significant change was found in acids. However, differences in main volatile compounds between young liquor and pressurized liquors disappeared after two to six months of storage. Principal component analysis (PCA) performed well in presenting overall differences among all liquor groups, which verified our previous findings in GC analysis. This research broadened the knowledge of HP processing on distilled alcoholic beverages and provided an alternative technique for liquor quality modification.

## Introduction

Chinese liquor is a popular alcoholic beverage with an annual consumption of four million kiloliters, the sale revenue of which reaches 75 billion dollars^1^. Typical manufacturing process of Chinese liquor generally involves fermentation, distillation, aging and, if required, blending procedures^2^. The newly distilled liquor (young, raw or fresh) often has some undesirable harsh flavor, and so they are usually aged in sealed pottery jars for several years to develop the bouquet flavor^3^. Therefore, the economic value of Chinese liquor is highly associated with its age since aging plays an indispensable role in producing high-quality liquors.

During natural aging process of Chinese liquor, many chemical reactions such as oxidation, esterification and hydrolysis can take place accompanied with the permeation and migration of some small and polar volatile compounds^4^. Recently, dozens of constituents have been extensively investigated and recognized as volatile compounds of distilled alcoholic beverages^5–7^. In relation to aging, Perestrelo *et al*.^8^ reported that acetal showed the highest and positive relationship with ages of Malvasia and Bual wines whereas volatile phenol showed the lowest correlation, specifically, 3-furfural, pantolactone, trans-dioxane and 2, 2-diethoxyethanol might be suggested as potential aging markers of Malvasia and Bual wines. Xu *et al*.^9^ found that 13 compounds in Chinese liquor including acids, alcohols, esters, aldehydes and furans decreased significantly during the first year of aging, and maintained at the same levels for the next three years. Besides, ethyl lactate was found to be the most stable volatile compounds during natural aging process.

As a non-thermal technology, high pressure (HP) has been widely used in food processing as it offers several advantages compared with conventional thermal techniques^10–14^. In addition, applications of HP treatment in wine and Chinese liquor have also been reported. Existing studies showed that HP treatments at higher conditions (650 MPa for at least 1 h) had immediate effect on physicochemical and sensorial characteristics of red wine^15^, while changes in wine which treated at lower HP conditions (400 to 500 MPa for 5 min) were perceptible only after several months of storage (had no immediate effect)^16,17^. However, immediate changes were observed in Chinese liquor even after lower conditions of HP treatments in our previous study^18^. The results showed that HP treatments at 300 MPa & 400 MPa caused significant decreases in alcohol content and total acid content of sauce-flavor Chinese liquor, meanwhile, increasing trends in total ester content and total solid content were also observed. Moreover, pressurized liquors scored higher than control group (without HP treatment) in sensory evaluation tests. These findings were in agreement with those in natural aging process. However, effects of high pressure treatment and the following storage process on main volatile compounds of Chinese liquor are still largely unknown.

In this study, gas chromatography with direct injection method was used for the identification and quantitation of main volatile compounds of Chinese liquor. After HP treatment, a more specific short term storage test (two to six months) was included to investigate the stability of the impact of HP treatment. The main objective of this research were to lay a theoretical foundation for the effect of HP treatment and short term storage strategy on changes in main volatile compounds of Chinese liquor, and to explore the possibility of HP to be used as a new technology for accelerating the aging of Chinese liquor from volatile compounds perspective.

## Results

### Identification and quantification analysis

**Figure 1:**
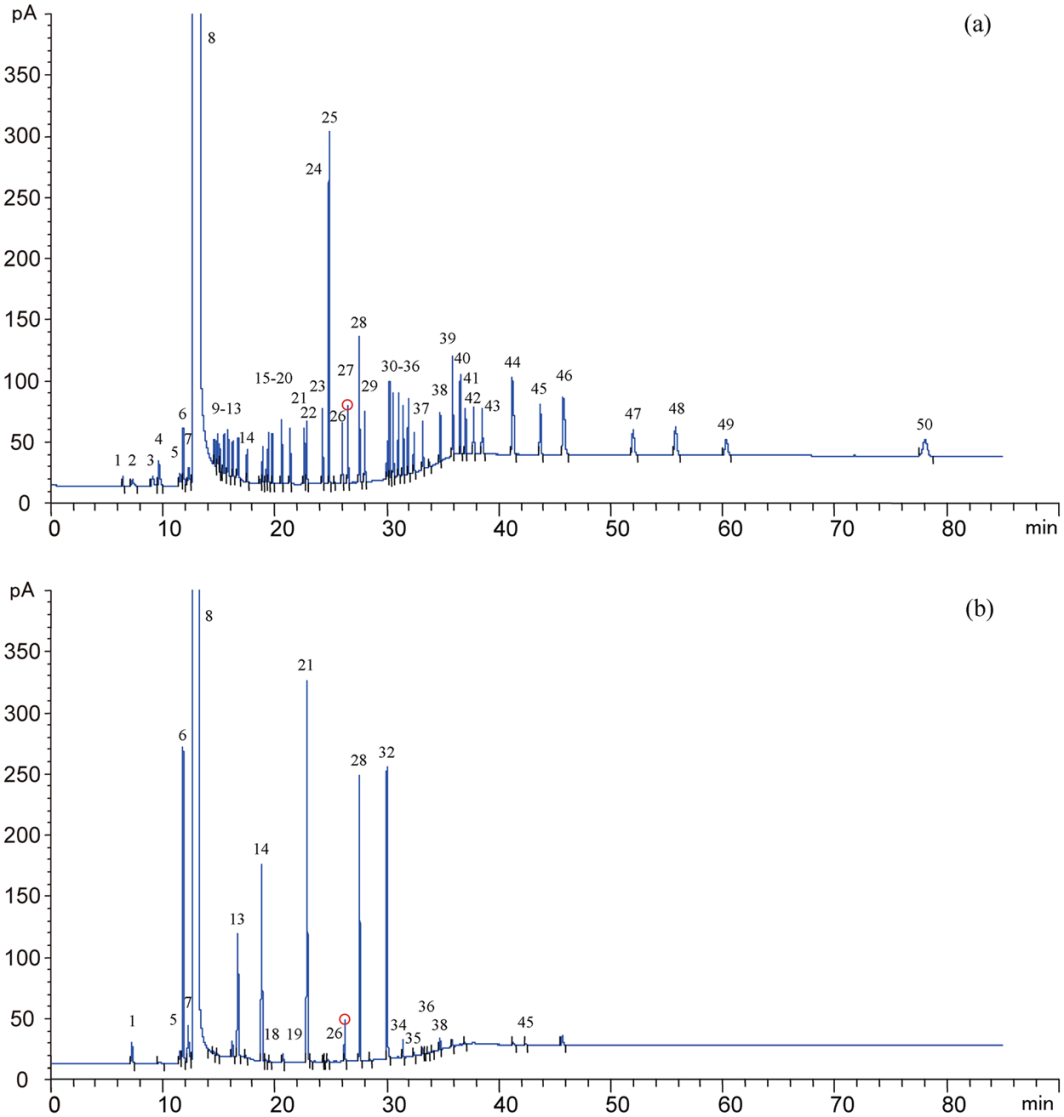
Typical chromatograms of mixed standard solution (**a**) and liquor sample (**b**). All compounds were numbered according to Table 1. Red circle: Amyl acetate (internal standard).

**Figure 2:**
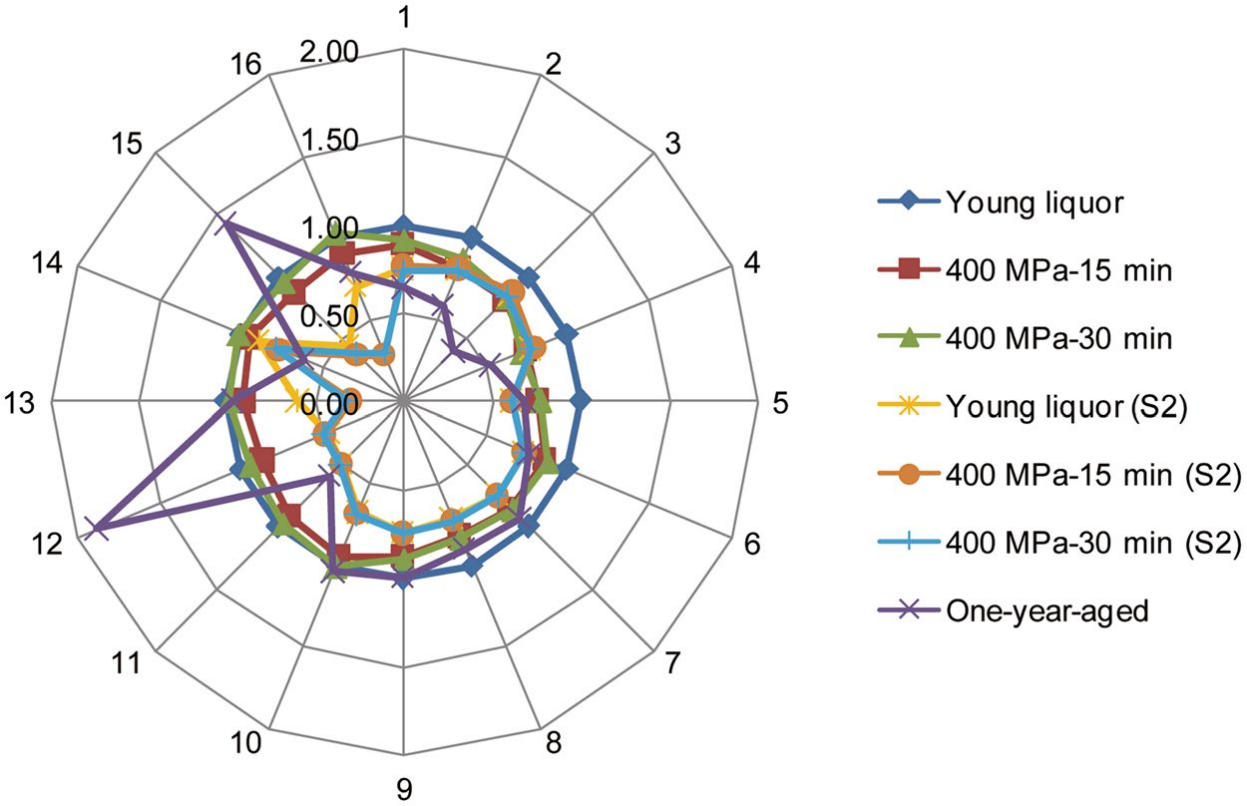
Radar map based on main volatile compounds. Acetaldehyde (1), Methanol (2), Ethyl acetate (3), Acetal (4), 2-Butanol (5), 1-Propanol (6), Isobutanol (7), n-Butanol (8), Isoamylol (9), Ethyl lactate (10), Acetic acid (11), Furfural (12), Propionic acid (13), Isobutyric acid (14), Butanoic acid (15), Isovaleric acid (16). (S2): Stored for two months.

Typical chromatograms of mixed standard solution and liquor samples are shown in Fig. 1a,b, all compounds were numbered according to Table 1. For liquor samples, 21 compounds were found to be abundant and dominant constituents in this type of Chinese liquor. Considering chemical groups of acids, alcohols, esters, aldehydes and furans, quantification results of 21 compounds are presented in Table 2. A radar map was used to visualize the general variation of main volatile compounds during natural aging and artificial aging (HP treatment and short term storage) processes. As shown in Fig. 2, concentration of each compound in young liquor was set as 1, and the relative concentration of each compound in other six groups was calculated by the actual concentration of the compound versus corresponding concentration in young liquor. 16 compounds were used since the other 5 compounds were only detected in one-year group.

**Table 1:** Composition of mixed standard solution.

Number	Compounds	Concentration	Number	Compounds	Concentration
1	Acetaldehyde	606.0	26	Ethyl lactate	916.7
2	Propanal	367.2	27	Ethyl oenanthate	363.8
3	Acetone	440.9	28	Acetic acid	1548.2
4	Ethyl formate	285.7	29	Trimethyl pyrazine	662.8
5	Methanol	318.0	30	n-Heptanol	412.5
6	Ethyl acetate	325.0	31	Ethyl caprylate	546.2
7	Acetal	646.0	32	Furfural	540.9
8	Ethanol	Solvent	33	Iso-amyl caproate	385.7
9	Ethyl propionate	298.6	34	Propionic acid	388.4
10	Diacetyl	356.9	35	Isobutyric acid	140.3
11	Ethyl isobutyrate	326.1	36	Butanoic acid	120.4
12	2-pentanone	401.1	37	Fuefuryl alcohol	456.0
13	2-Butanol	358.4	38	Isovaleric acid	136.4
14	1-Propanol	426.3	39	Ethyl caprate	450.0
15	Ethyl butyrate	127.3	40	Diethyl succinate	672.1
16	Butyl acetate	336.2	41	Valeric acid	395.8
17	2-Hexanone	357.7	42	Ethyl phenylacetate	653.8
18	Isobutanol	1092.3	43	Hexanoic acid	412.2
19	n-Butanol	386.9	44	Ethyl lactate	536.8
20	Ethyl valerate	365.9	45	Phenylethanol	210.1
21	Isoamylol	1038.2	46	Heptylic acid	502.9
22	n-Amyl alcohol	395.8	47	Octanoic acid	447.3
23	Ethyl hexanoate	298.0	48	Ethyl myristate	496.8
24	Hexyl acetate	376.2	49	n-Nonoic acid	512.2
25	2-Hydropxyheptane	365.4	50	Ethyl palmitate	460.2

Concentration: mg/L.

**Table 2:** Main volatile compounds of liquor samples with different treatments.

Number	Compounds	Young	400-15	400-30	Young(S2)	400-15(S2)	400-30(S2)	One year
1	Acetaldehyde	237.81 ± 11.87a	212.50 ± 12.72b	217.75 ± 10.66b	178.89 ± 6.97c	181.42 ± 12.49c	176.52 ± 9.66c	152.99 ± 12.67d
2	Methanol	79.79 ± 3.46a	65.10 ± 1.89c	70.42 ± 2.12b	64.37 ± 2.39c	64.78 ± 2.50c	64.28 ± 2.69c	46.75 ± 2.06d
3	Ethyl acetate	1741.48 ± 87.52a	1418.78 ± 85.08b	1450.86 ± 76.20b	1472.49 ± 69.85b	1524.67 ± 88.60b	1454.40 ± 59.43b	694.23 ± 36.17c
4	Acetal	519.72 ± 30.60a	390.36 ± 25.19b	378.95 ± 28.88b	402.45 ± 25.46b	416.49 ± 24.61b	401.96 ± 30.19b	275.31 ± 22.76c
5	2-Butanol	29.97 ± 1.35a	22.65 ± 1.04b	23.21 ± 1.76b	18.30 ± 1.88c	18.41 ± 1.96c	18.36 ± 2.47c	20.54 ± 2.54c
6	1-Propanol	338.84 ± 16.54a	291.73 ± 13.38b	296.90 ± 20.40b	248.92 ± 9.67c	250.50 ± 15.25c	250.29 ± 16.79c	261.24 ± 20.35c
7	Ethyl butyrate	ND	ND	ND	ND	ND	ND	4.28 ± 0.53
8	Isobutanol	637.67 ± 39.72a	545.46 ± 20.09b	553.07 ± 38.16b	477.81 ± 18.13c	480.14 ± 25.44c	479.77 ± 28.13c	593.72 ± 29.01a
9	n-Butanol	13.84 ± 1.73a	11.37 ± 1.26a	11.60 ± 1.19a	9.84 ± 1.64a	10.13 ± 1.57a	10.07 ± 1.98a	12.53 ± 1.79a
10	Isoamylol	800.53 ± 41.79a	697.90 ± 44.61b	710.83 ± 45.37b	594.50 ± 38.17c	597.56 ± 37.39c	598.68 ± 40.96c	793.02 ± 38.19a
11	Ethyl hexanoate	ND	ND	ND	ND	ND	ND	52.82 ± 4.69
12	Ethyl lactate	446.38 ± 30.40a	423.64 ± 20.71a	451.06 ± 28.87a	302.33 ± 17.52b	306.78 ± 14.99b	309.78 ± 12.05b	465.10 ± 31.16a
13	Ethyl oenanthate	ND	ND	ND	ND	ND	ND	14.43 ± 1.62
14	Acetic acid	1194.00 ± 91.82a	1095.88 ± 86.71a	1158.02 ± 79.46a	592.01 ± 41.40c	599.30 ± 40.80c	605.75 ± 38.26c	701.73 ± 40.19b
15	Furfural	19.17 ± 1.09b	16.65 ± 1.88b	18.09 ± 1.62b	8.82 ± 1.03c	9.21 ± 0.96c	9.36 ± 1.41c	36.05 ± 2.56a
16	Propionic acid	7.83 ± 0.57a	7.11 ± 0.69a	7.79 ± 0.77a	4.71 ± 0.62b	2.35 ± 0.55c	2.48 ± 0.41c	7.61 ± 0.41a
17	Isobutyric acid	14.95 ± 1.92a	14.17 ± 2.04a	15.09 ± 2.27a	13.27 ± 2.41a	11.46 ± 2.07a	11.69 ± 1.89a	9.18 ± 0.60b
18	Butanoic acid	20.87 ± 1.56b	18.20 ± 1.71b	20.01 ± 2.07b	9.12 ± 0.79c	7.84 ± 0.86c	8.11 ± 0.91c	29.69 ± 2.01a
19	Isovaleric acid	3.62 ± 0.26a	3.28 ± 0.30a	3.73 ± 0.29a	2.55 ± 0.21b	1.02 ± 0.16c	1.07 ± 0.15c	2.87 ± 0.18b
20	Phenylethanol	5.90 ± 0.44b	4.43 ± 0.56b	5.48 ± 0.69b	ND	ND	ND	11.59 ± 0.68a
21	Ethyl palmitate	ND	ND	ND	ND	ND	ND	13.09 ± 0.95

All values are expressed as means (mg/L) ± standard deviation (SD).

Different letters indicate significant differences (p < 0.05).

ND: Not detected.

(S2): Samples stored for two months.

### Changes in main volatile compounds during natural aging process

In order to evaluate the aging effect of HP treatment and following short-term storage on Chinese liquor, changes in main volatile compounds during natural aging process were firstly characterized. As can be seen from Table 2, 17 compounds were detected in young liquor while 21 were found in one-year-aged liquor. Results showed that natural aging process significantly affected all studied chemical groups, especially for acids and esters.

Acids in Chinese liquor are products of sugar oxidation or of alcoholic fermentation during liquor making process. Acetic acid was found to be the most abundant compound in this group with the concentration of 1194 mg/L in young liquor, which was in agreement with previous studies^9,19^. After one year of aging, decrease in acetic acid content was observed from 1194 mg/L to 701 mg/L (p < 0.05), which might have been caused by the volatilization through the jar and the esterification with alcohols during natural aging process. This decrease trend could conduce to produce high-quality Chinese liquor, since acetic acid contributing negatively to the liquor bouquet^4^. In addition, a slight increase (p < 0.05) in butanoic acid content whereas decreases (p < 0.05) in isobutyric acid and isovaleric acid contents were observed, but no significant change was found in propionic acid content. Acids affect the taste and mouth-feel of liquors, increase color stability, reduce oxidation and together with ethanol, are largely responsible for the microbial and physicochemical stability of liquors^20^.

In esters group, ethyl acetate and ethyl lactate were the most prominent representatives, with concentrations of 1741 and 446 mg/L in young liquor, respectively. A decrease of 60% (p < 0.05) was observed in ethyl acetate content after one year of aging, from 1741 mg/L to 694 mg/L. Ethyl acetate has a significant effect on organoleptic characteristics of wines and distilled liquors, depending on its concentration^21^, namely “fingernail polish remover” properties at high concentration whereas fruity properties at low concentration^22,23^. In addition, long-chain esters, such as ethyl butyrate, ethyl hexanoate, ethyl oenanthate and ethyl palmitate were detected in one-year-aged liquor. In the manufacturing procedures of Chinese liquor, unlike ethyl acetate, long-chain esters are mainly generated from moderate esterification reactions between alcohols and corresponding acids during aging rather than fermentation and distillation, thus they gradually formed after one year of aging. Moreover, there might be very small amounts of long-chain esters in young liquor which was much lower than the detection limit and could not be detected in this research. It seemed that ethyl lactate was extremely stable during aging process as no significant difference was observed between young and aged liquors (at least during the first year of aging).

As for alcohols, isobutanol, n-butanol and isoamylol were kept at the same levels (p > 0.05), while decreasing trends (p < 0.05) were observed in methanol, 2-butanol and 1-propanol. Meanwhile, a slight increase was found in phenylethanol content (p < 0.05) after one year of aging. It is recognized that there will be some decline in alcohols content during natural aging process of liquors, and this has been linked to changes in the structure of water and alcohols molecules^24^.

Acetaldehyde and acetal are the most toxic metabolite created by alcohol metabolism originating from fermented raw materials^25^. Therefore, significant decreases found in acetaldehyde and acetal contents during the first year of aging reduces the potential toxicity (caused by acetaldehyde and acetal) of Chinese liquor. Besides, the accumulation of furan as presented in Table 2 is usually considered as an aging marker as it can be formed by pyrolysis of carbohydrates, dehydration of sugars through Maillard reaction, and caramelization, which occurs during fermentation and aging process^26^.

### Impact of HP treatments on main volatile compounds

It can be obviously observed from Table 2 that the HP treatments significantly affected some chemical groups of pressurized liquor, especially for alcohols and aldehydes when compared with young liquor.

As can be seen, acetic acid, propionic acid, isobutyric acid, butanoic acid and isovaleric acid contents maintained at the same level (p > 0.05) after HP treatments. Similar phenomenon was also observed in esters group. Though ethyl lactate content was slightly altered after treatment, differences were not statistically significant. What’s more, ethyl butyrate, ethyl hexanote, ethyl oenanthate and ethyl palmitate were not detected neither in young liquor nor in pressurized liquors. Only ethyl acetate was significantly affected by HP treatments, with decreasing rates of 19% and 17% at conditions of 400 MPa-15 min and 400 MPa-30 min, respectively. These results, however, contradicted against our previous findings, in which we stated that HP treatments resulted in a significant increase of ethyl acetate content in sauce-flavor Chinese liquor^18^. This may has been caused by the differences in volatile composition of these two types of Chinese liquor (sauce-flavor & light-flavor), and the chemical reaction equilibrium under HP conditions could be affected in different ways. However, more studies are needed to have a better understanding of the reaction mechanism of compounds in Chinese liquor under high pressure conditions so that we can make it clear which particular chemical reaction equilibrium has been affected. Results from Table 2 also revealed significant reduction in alcohol group except for n-butanol and phenylethanol after HP treatments; however, differences were still existed in comparison with one-year-aged liquor. As for aldehydes group, acetaldehyde and acetal contents were significantly (p < 0.05) decreased after HP treatment, which was in consistent with natural aging process. Furan maintained at the same level as no significant difference was observed between young liquor and pressurized liquors. Though the mechanism of how HP treatments affect volatile compounds of Chinese liquor is still unknown, these changes have been explained by the principle of Le Chatelier which states that any phenomenon accompanied by a decrease in reaction volume is enhanced by an increase in pressure, and vice versa^27^. Thus chemical reaction equilibrium in Chinese liquor can be altered by HP treatments, which resulted in changes in volatile composition. Furthermore, the decrease trends of some compounds might have been caused by the enhanced volatilization and hydration under HP conditions.

In general, changes in alcohols and aldehydes after HP treatments were in consistent with those in natural aging process, as can be seen intuitively in Fig. 2. However, HP treatments failed to alter contents acids and long-chain esters in this research. In addition, no significant difference was found between two selected HP conditions (400 MPa-15 min and 400 MPa-30 min). It should be noted that despite some differences were found in main volatile compounds of the pressurized liquors in comparison with young liquor, big overall gaps were still existed between pressurized and one-year-aged liquors (Fig. 2).

### Impact of following two months of storage on main volatile compounds

Previous studies conducted by Santos *et al*. elucidated that HP treatments with processing time around 5 min and pressures between 400 MPa and 500 MPa affected volatile compounds of white and red wine, however, the effect was only perceptible after several months of storage, changing aroma characteristics of the wine^16^. The results led us to hypothesize that HP treatment assists in accelerating changes in volatile compounds of Chinese liquor and the effect can be enlarged during the following storage. As a control group, young liquor was also stored for two months under the same condition with pressurized liquors.

Results from Table 2 showed that after two months of storage, few differences were observed among the volatile compositions of three storage groups, young (S2), 400-15 (S2) and 400-30 (S2). Ethyl lactate contents in three groups were even much lower than the one in one-year-aged group, indicating an increase trend during the following storage process. A slight reduction was observed in ethyl acetate content of young liquor while no significant change was found in pressurized liquors. And the short term storage still had no effect on long-chain esters; in fact, these long-chain esters were detected only after one year of aging. As for acids group, as can be seen from Table 2, 4 acids decreased (p < 0.05) while isobutyric acid stayed at the same level (p > 0.05) after two months of storage. As the dominant compound in this group, a sharp decline from 1100 mg/L to 600 mg/L was observed in acetic acid, which was also lower than the one in one-year-aged liquor (700 mg/L). It can be inferred that as a kind of volatile organic acid with short carbon chain, the initial decline of acetic acid might have been caused by the volatilization during the storage of first two months; after that, the concentration of acetic acid increased gradually with the decomposition of ethyl acetate during the following storage process, since ethyl acetate was found to be decreased dramatically during the first year of aging. Similar anomalous changes were also observed in other compounds, to be more specific, concentrations of furfural and almost all alcohols after short term storage were all beyond the range of young liquor and one-year-aged liquor (Fig. 2), which was unexpected.

Based on these findings, we presumed that the major factors which have effect on volatile compounds of Chinese liquor are changing with the storage time during the natural aging process. As stated before, volatilization plays a more important role in the initial storage process, causing decreases in most of the compounds in the first two months. As the storage time goes on, volatile compounds are mainly affected by a series of complex chemical reactions, which are catalyzed by metallic substances exists in pot used for liquor storage, as a consequence, after two month of storage, some compounds decrease further while others increase on the contrary during the following storage process till one year. That could be the reason for anomalous changes observed in some volatile compounds of Chinese liquor during storage process; however, this hypothesis has not been verified by any research so far. Moreover, the decrease trends found in acetaldehyde and methanol contents were in consistent with the natural aging process (Fig. 2).

Above all, short term storage failed to enlarge the differences in volatile compounds between young and pressurized Chinese liquor, on the contrary, the initial impact of HP treatments disappeared after two months of storage, which was unexpected. It seemed that storage played a more important role than HP treatments in changes of volatile composition of Chinese liquor during this process. These findings were paradoxical with former studies as presented before^16,17^. In addition, the anomalous changes found in some of the compounds suggested the existence of complex chemical reactions during the storage process of Chinese liquor.

### Principal component analysis (PCA) based on 7 groups

**Figure 3:**
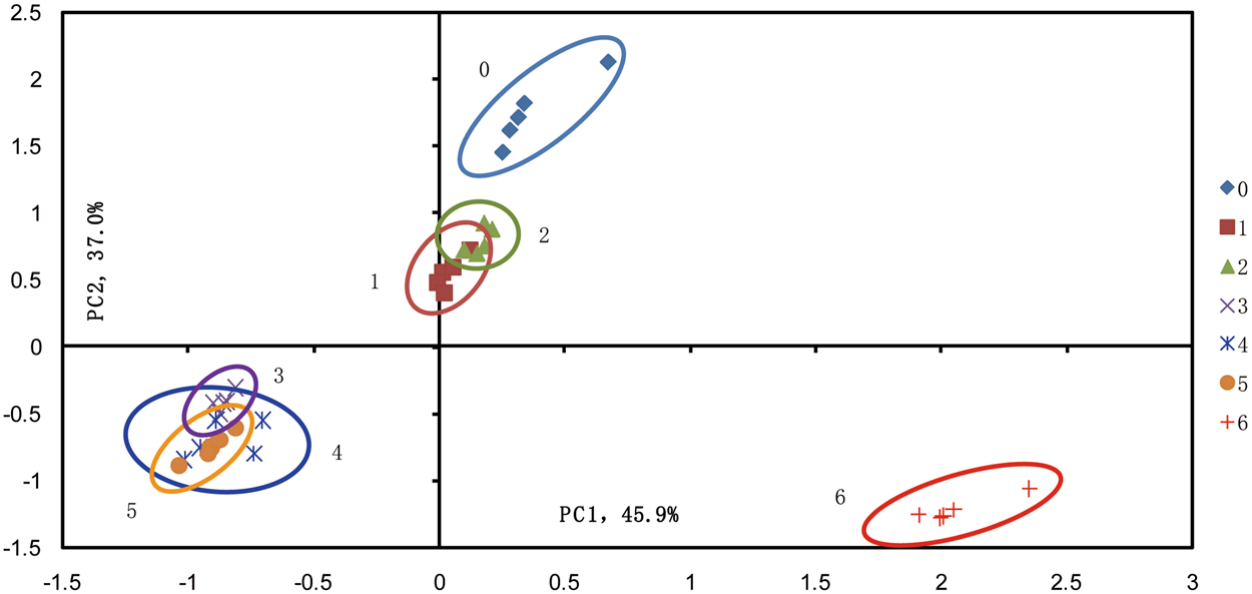
Scores plot of PCA based on main volatile compounds. Young liquor (0), 400 MPa-15 min (1), 400 MPa-30 min (2), Young liquor stored for two months (3), 400 MPa-15 min stored for two months (4), 400 MPa-30 min stored for two months (5), One-year-aged liquor (6).

As presented in Fig. 3, scores plot was used for studying the character location of Chinese liquor with different treatments and evaluating overall differences of volatile composition among all groups. As can be seen, the first principal component (PC1) and the second principal component (PC2) were taken as coordinate axes, which explained 82.85% of the total variance of all compounds. In PCA, the higher cumulative contribution rate is and the more original information will be reflected^28^. Generally, first and second principal component are regarded as good representative of original information, when the accumulated variance exceed 80%.

As can be seen, liquor samples were located in three different quadrants and different groups were well-separated from each other. Young liquor and pressurized liquors were distributed in the first quadrant and located closely with each other, which verified the conclusion as stated before, despite some differences were observed in volatile compounds of the pressurized liquors in comparison with young liquor without treatment, the impact of HP treatments was still minimal. Liquor samples with two months of storage (young and pressurized liquors) were clustered as one category in the third quadrant, signifying the disappearance of initial difference in volatile compounds between young and pressurized liquors. One-year-aged liquors were isolated in the fourth quadrant, the relative long distance as showed in the coordinate system between one-year-aged group and other groups indicated that overall gaps still existed between artificial aged liquors at selected conditions (HP treatments and short storage) and natural aged liquors even only after one year of aging.

### Six months of storage

The results were unexpected since this work was carried out on the hypothesis that HP treatment assists in accelerating changes in volatile compounds of Chinese liquor based on previous studies^17,18^. Hence the experiments were repeated again at six months of storage. The additional results for six months of storage were almost identical (Table S1) to the one presented for the two months of storage, reconfirming the conclusions from the preceding section that differences between young liquor and pressure treated liquors found immediately after the treatment gradually disappeared within a short to medium term of storage. The variation trends of main volatile compounds detected in three groups (stored for six months) were in consistent with each other during the whole storage process. As can be seen from Table S1, some compounds were kept at the same level (acetaldehyde, methanol, 2-butanol, 1-propanol, ethyl butyrate, n-butanol, isovaleric acid, phenylethanol, ethyl palmitate), while others were slightly decrease (ethyl acetate, acetal, isobutanol, isoamylol, ethyl lactate, acetic acid), in addition, increase trends in furfural and long-chain esters (ethyl hexanoate, ethyl oenanthate) were observed as compared with two months stored liquors. However, the sophisticated chemical reactions which result in these changes during the storage process are still unknown.

## Conclusions

This research confirmed the possibility of using HP to alter the volatile composition of Chinese liquor, however, it can be hard for this single technique to be used as a new technology for shortening the natural aging period of young liquor at least from the volatile compounds perspective, since the initial differences in main volatile compounds caused by HP gradually disappeared after two to six months of storage. Our new findings enriched the theoretical basis of the impact of HP treatments on main volatile compounds of Chinese liquor. Based on our results, further studies should be conducted to have a better understanding of the mechanism of the chemical reactions which take place during HP treatment and storage process.

## Materials and Methods

### Chinese liquor samples and chemicals

Chinese liquor “*Junchang*” (light-flavor) was provided by a local liquor factory in Sichuan province, and stored at room temperature before use. Young liquors without storage and one-year-aged liquors were used. Young liquors (15 samples) were randomly separated into two sub-groups, one (5 samples) together with one-year-aged liquor (5 samples) was regarded as control group, and the other one (10 samples) was subjected to HP treatments for subsequent use. A mixed standard solution (Donglilong Information Technology Co. Ltd., Lanzhou, China) special for Chinese liquor gas chromatography analysis was used for identification and quantitation analysis. As shown in Table 1, this mixed standard solution contained 50 compounds, which covered almost all the main volatile compounds of Chinese liquor. In order to ensure the accuracy of quantitation results, amyl acetate was used as internal standard substance. All groups were analyzed both before and after storage (two and six months).

### High pressure treatments and storage

HP treatments were carried out using laboratory scale high pressure equipment. The system consisted of a HP unit (UHPF-750, 5 L, Kefa, Baotou, China), was equipped with K-type thermocouples (Omega Engineering, Stamford, CT, USA) and a data logger (34970 A, Agilent Technologies GMBH, Germany) for temperature measurement and a thermostat jacket connected to a water bath (SC-25, Safe, China) for maintaining the processing temperature. The intensifier used for generating the pressure was a batch type unit which built-up the pressure in a stepwise ladder-like process. Water was used as pressure transmitting medium (PTM) in this study, and the pressure vessel was maintained at 25 °C before pressurization. Sample temperature was monitored during the tests and was recorded at 1 s interval. Normally sample temperature is expected to increase by 3 °C every 100 MPa pressure rise due to adiabatic compression. However, to minimize this adiabatic heating, a low rate of pressurization was maintained (~100 MPa/min) so that the sample temperature easily equilibrated to the set point temperature of 25 °C. The pressure release time was kept less than 5 s.

Sufficient young liquors were packaged with polyethylene terephthalate (PET) bags and sealed by a plastic-envelop machine. After that, young liquors were treated by HP at conditions of 400 MPa-15 min and 400 MPa-30 min. Each condition was carried out with five replicates (five liquor samples for each condition). After HP treatments, ten liquor samples were individually transferred into ten pottery jars (500 mL for each pottery jar) for subsequent use. All the pottery jars were placed under the condition of protection from light at ambient temperature (around 25 °C) to imitate the natural aging process of Chinese liquor.

### Gas chromatography analysis

5 samples from young group, 5 samples from one-year group, 5 samples from 400 MPa-15 min group (immediately after HP treatment) and 5 samples from 400 MPa-30 min group (immediately after HP treatment) were firstly analyzed using gas chromatography to characterize the volatile composition of all groups. In order to evaluate the effect of short-term storage on volatile composition of HP treated liquors, GC tests were also carried out on ten pressurized samples (5 for 400 MPa-15 min and 5 for 400 MPa-30 min) after two and six months of storage. The amount of liquor sample in each pottery jar was about 500 mL and only about 30 mL of the sample was used for each batch of GC test with 5 replicates for each sample, the rest of the sample was sealed and stored in the jar (Fig. S1). The whole process from open to seal was less than 20 seconds, which was even shorter than the subsequent filtering procedure, thus, it had little effect on the GC results and on the volatile composition of the rest of the sample.

Gas chromatography analysis was performed using an Agilent 7890 A gas chromatography (GC) system equipped with a flame ionization detector (FID). The column carrier gas was nitrogen at constant flow rate of 1 mL/min. All samples were analyzed on a LZP-950 column (50 m × 0.32 mm i.d., 1.0 μm film thickness). First of all, liquor samples were filtered into a 2 mL autosampler vial using a filtering membrance with the pore diameter of 0.45 μm to remove impurities. Then a 1 μL of filtered sample was injected into GC from the autosampler vial, and the split ratio was 1:1. The oven temperature was held at 65 °C for 8 min, then raised to 200 °C at a rate of 5 °C/min, and held at 200 °C for 50 min; injector and detector temperature were 230 °C and 250 °C, respectively.

The identification analysis was made by comparing the retention times of all compounds in mixed standard solution (Fig. 1a) with those in liquor sample (Fig. 1b) based on the theoretical foundation which states that a certain substance has a certain retention time under the same chromatographic condition. The quantification of volatile compounds in Chinese liquor was realized using calibration curves. In addition, amyl acetate was added into samples as internal standard substance, and the final quantification results were corrected using this internal standard (350 mg/L in 1 mL of liquor sample). All liquor samples were analyzed by direct injection method, replicated in five times and values were averaged.

### Statistical analysis

Data of all groups obtained from GC analysis were firstly processed by One Way ANOVA at the 5% significance level using IBM SPSS statistics 21.0 to compare the statistical differences. Then PCA was applied to reduce the dimensionality and complexity of original data matrix while retained the maximum amount of variability^29^ and to evaluate the overall differences of volatile compounds among all liquor groups with different treatments by deriving the first two principal components from compounds data. PCA was also conducted with IBM SPSS statistics 21.0.

## Electronic supplementary material

Dataset 1
