# Supplementary material for: Effect of high pressure treatment and short term storage on changes in main volatile compounds of Chinese liquor
Source: Sci Rep. 2017 Dec 8;7:17228. doi: 10.1038/s41598-017-17549-x (PMC5722930; doi:10.1038/s41598-017-17549-x)
Supplement: Supplementary file 1 — Dataset 1 [file 41598_2017_17549_MOESM1_ESM.docx]

**Effect of high pressure treatment and short term storage on changes in main volatile compounds of Chinese liquor**

Menglong Xu ^ab^, Songming Zhu ^ab^, Hosahalli S. Ramaswamy ^c^, Yong Yu ^ab*^

^a^ *College of Biosystems Engineering and Food Science, Zhejiang University, Hangzhou 310058, China.*

^b^ *Key Laboratory of Equipment and Informatization in Environment Controlled Agriculture, Ministry of Agriculture, Hangzhou 310058, China.*

^c^ *Department of Food Science, McGill University, St-Anne-de-Bellevue, QC H9X 3V9, Canada.*

* **Corresponding author**. Tel.: +8613018935120; fax: +860571-88982181.

E-mail address: yyuzju@zju.edu.cn (Yong Yu)


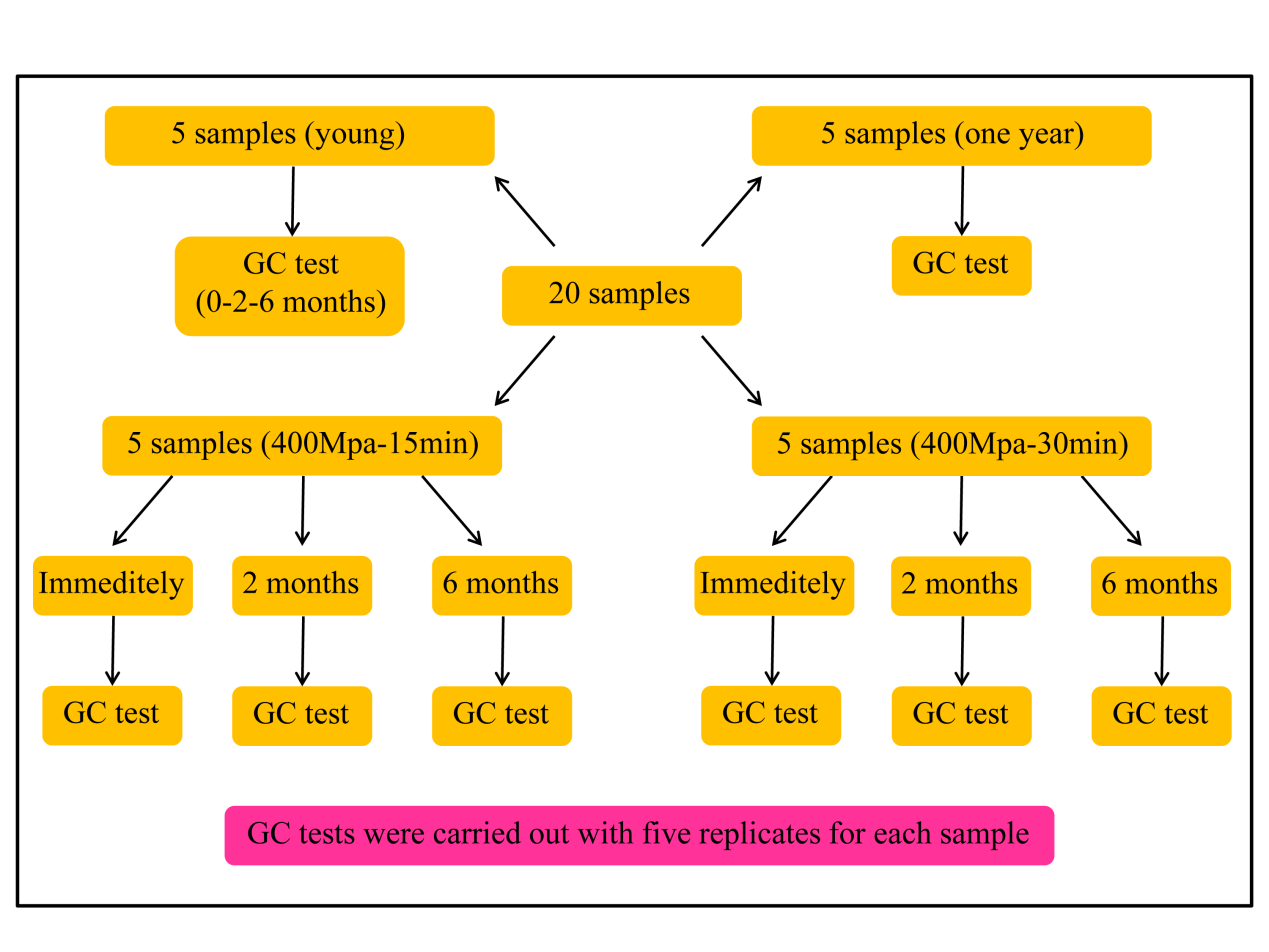

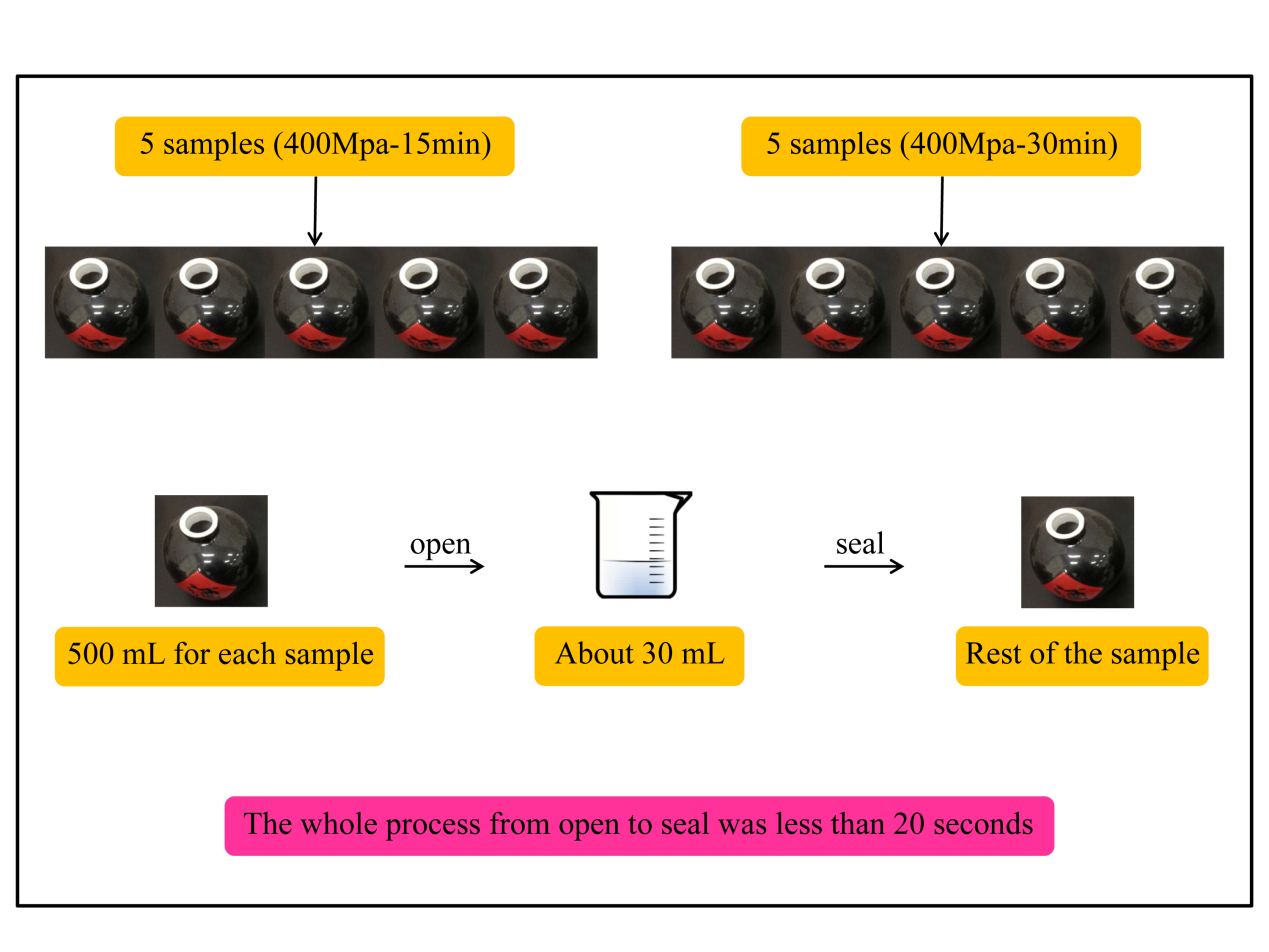


Fig. S1. Experimental design

Table S1 Main volatile compounds of Chinese liquor after six months of storage

| **Number** | **Compounds** | **Young (S6)** | **400-15 (S6)** | **400-30 (S6)** |
| --- | --- | --- | --- | --- |
| 1 | Acetaldehyde | 170.56±8.22a | 172.34±9.61a | 168.52±7.66a |
| 2 | Methanol | 58.61±3.25a | 59.11±2.97a | 59.90±2.43a |
| 3 | Ethyl acetate | 997.19±39.69a | 1021.63±40.58a | 995.24±35.03a |
| 4 | Acetal | 352.07±19.07a | 356.65±21.19a | 362.71±18.70a |
| 5 | 2-Butanol | 19.26±2.57a | 20.33±1.98a | 20.16±2.21a |
| 6 | 1-Propanol | 257.55±13.60a | 262.49±15.23a | 255.16±16.93a |
| 7 | Ethyl butyrate | ND | ND | ND |
| 8 | Isobutanol | 449.58±32.01a | 453.92±29.61a | 461.88±35.72a |
| 9 | n-Butanol | 11.55±2.17a | 10.97±1.95a | 10.89±2.02a |
| 10 | Isoamylol | 570.30±40.25a | 562.98±38.06a | 575.29±35.67a |
| 11 | Ethyl hexanoate | 19.18±2.01a | 18.92±1.95a | 18.69±1.80a |
| 12 | Ethyl lactate | 286.71±18.76a | 279.92±19.05a | 288.63±21.22a |
| 13 | Ethyl oenanthate | 6.51±0.96a | 6.67±0.89a | 6.38±0.77a |
| 14 | Acetic acid | 556.27±32.91a | 549.65±28.72a | 561.90±27.21a |
| 15 | Furfural | 20.65±1.67a | 19.91±1.73a | 20.05±1.92a |
| 16 | Propionic acid | 5.32±0.55a | 5.18±0.49a | 5.51±0.43a |
| 17 | Isobutyric acid | 11.91±0.36a | 11.80±0.28a | 11.96±0.31a |
| 18 | Butanoic acid | 11.26±0.94a | 11.58±1.01a | 10.96±0.79a |
| 19 | Isovaleric acid | 1.57±0.22a | 1.61±0.18a | 1.60±0.19a |
| 20 | Phenylethanol | ND | ND | ND |
| 21 | Ethyl palmitate | ND | ND | ND |

All values are expressed as means (mg/L) ± standard deviation (SD)

Different letters indicate significant differences (p < 0.05).

ND: Not detected.

(S6): Samples stored for six months.
